# Journal impact factor is NOT a measure of scientific or social worth of an article

**DOI:** 10.1093/aesa/saad041

**Published:** 2024-01-03

**Authors:** David W Onstad

**Affiliations:** Editor-in-Chief

In the 21st century, citations and the related journal impact factor (JIF) have become like cryptocurrency. Entomologists and their supervisors use these calculated numbers as an easy quantifier for scientific contributions versus the more traditional methods such as number of published, peer-reviewed articles, or even qualitative estimates of value in research. In a future editorial, I will address some major ethical and sociological issues concerning reliance on citations and the JIF. Here I simply provide 3 examples that question the value of relying on the JIF as a measure of scientific worth.

The JIF, as calculated by Clarivate Analytics, is a measure of the average number of times articles from a 2-yr time frame have been cited in a given year, according to citations captured in the Web of Science database. The JIF for year *T* equals *A*/*B*, where *A* is the number of times articles published during the prior 2 yr, *T* − 2 to *T* − 1, were cited in indexed journals during year *T*, and *B* is the total number of articles from the journal published in those 2 y. The JIF is announced in year *T* + 1.

Most entomologists would agree that avocado entomology is an important area of study. Avocados are a $12 billion industry. Not a major crop but a product worthy of our attention. Avocado entomology exemplifies small-scale science important to its stakeholders and entomology. What would be the value of research in this field according to the JIF?

Example 1. In December 2023, I searched the bibliographic database CAB Abstracts using the topics avocado and “insect OR mite.” Good papers are published in the entomology journals and some are highly cited (Table 1). No hits were obtained in a search with *PNAS* or *Science* as the journal. There seems to be no reason why an author, who has a scientific contribution about avocado entomology, would choose to publish a paper in *Science*, *PNAS*, or *Nature* for JIF prestige.

**Table 1.** Number of papers published in journals about avocado and arthropods

			Citations per paper	
Journal	JIF	Number of papers	Median	Range
Nature	70	1	NA	10
Annals	2	18	15	0–115
JEE	2	141	9	0–133
EnvEnt	2	12	11	1–32

*Annals of the Entomological Society of America*; *Journal of Economic Entomology and Environmental Entomology*.

Example 2. In the spring of 2023, I searched all journals but restricted it to the past 5 yr in CAB Abstracts and searched for topics avocado and “insect OR mite.” There were 224 hits with a range of 0–27 citations per paper with a median citation of 1 per paper (Table 2). (I omitted 2 of original top 10 because the titles and abstracts indicated that arthropods were minor subjects of the papers.)

**Table 2.** Data for 10 of top 12 papers captured in search of CAB Abstracts for topics “avocado and insects OR mites” over past 5 yr

	5 yr	Avocado		General
Journal	JIF	in title?	Citations	Subject
NR^a^	4.1	Yes	27	*D. melanogaster*
PRSB^a^	6.0	No	25	Pollinators
Mycologia	3.3	No	23	*Fusarium* and *Euwallacea*
SciRep	5.5	No	16	Pollinators
JApplEnt	2.4	Yes	14	Avocado pollinator review
SciRep	5.5	Yes	14	*Xyleborus* IPM
JEE	2.5	No	14	*Xyleborus glabratus*
AgForEnt	2.1	Yes	14	*Euwallacea* dispersal
JPestSci	5.6	No	14	*Euwallacea* and volatile lures
PLoS One	NA^b^	Yes	12	*Scirothrips perseae*

^a^NR is Neurochemical Research; PRSB is Proc Royal Society B.

^b^Not reported.

Those papers with pollinators as a focus (including the only review) have a mean citation rate of 18; the rest have mean rate of 19 (Table 2). Those concerning Ambrosia beetles (*Euwallacea* and *Xyleborus (Scolytinae)*) averaged 16 citations per paper. The correlation coefficient for JIF and citations was 0.23 (*n* = 9). For the 5 focused on Ambrosia beetles, the correlation coefficient was −0.17. Note that the publisher of PLOS does not consider JIF to be a reliable or useful metric to assess the performance of individual articles. Again, it seems that entomologists in need of avocado entomology information who restrict publication searches to journals with high JIF would likely be missing important research results.

Example 3. JIFs, and associated reputations of both scientists and journals in which they publish, are determined by how many citations a journal article receives in the first 2 yr after publication. However, a 2023 assessment of ESA journals shows that many older papers are highly valued more than 20 yr after they are published. In terms of views of papers on the Internet, 4, 2, 2, and 2 out of the top 10 papers viewed in 2023 in *AESA*, *EE*, *JEE*, and *JME*, respectively, were actually published more than 20 yr before the assessment. Thus, at least 20%–40% of papers cannot be judged simply on the citations over first 2 yr after publication.

In a future editorial, I will discuss how citations and JIF are biased in favor of men, review papers, articles and subjects with many researchers, and papers concerning methods with wide applicability. The JIF was not created to be used in the scientific reward system, but rather a construct of publishers to compete. It does not represent a quantitative measure of the relative value of an author or a paper to society or science. Entomologists must not be influenced by JIF when they submit an article, read an article or judge one.

